# Diurnal variations of hormonal secretion, alertness and cognition in extreme chronotypes under different lighting conditions

**DOI:** 10.1038/srep33591

**Published:** 2016-09-20

**Authors:** L. Maierova, A. Borisuit, J.-L. Scartezzini, S. M. Jaeggi, C. Schmidt, M. Münch

**Affiliations:** 1 Ecole Polytechnique Fédérale de Lausanne, Solar Energy and Building Physics Laboratory, CH-1015 Lausanne, Switzerland; 2 Czech Technical University in Prague, UCEEB, Trinecka 1024, 273 43 Bustehrad, Czech Republic; 3 School of Education, University of California, Irvine, USA; 4 GIGA-CRC in Vivo Imaging, University of Liège, Belgium

## Abstract

Circadian rhythms in physiology and behavior are modulated by external factors such as light or temperature. We studied whether self-selected office lighting during the habitual waking period had a different impact on alertness, cognitive performance and hormonal secretion in extreme morning and evening chronotypes (N = 32), whose preferred bed- and wake-up times differed by several hours. The self-selected lighting condition was compared with constant bright light and a control condition in dim light. Saliva samples for hormonal analyses, subjective ratings of alertness, wellbeing, visual comfort and cognitive performance were regularly collected. Between the self-selected and the bright, but not the dim lighting condition, the onset of melatonin secretion in the evening (as marker for circadian phase) was significantly different for both chronotypes. Morning chronotypes reported a faster increase in sleepiness during the day than evening chronotypes, which was associated with higher cortisol secretion. Wellbeing, mood and performance in more difficult cognitive tasks were better in bright and self-selected lighting than in dim light for both chronotypes, whereas visual comfort was best in the self-selected lighting. To conclude, self-selection of lighting at work might positively influence biological and cognitive functions, and allow for inter-individual differences.

Diurnal changes in human physiology and behavior are driven by the endogenous masterclock in the suprachiasmatic nuclei of the hypothalamus^1^ as well as by peripheral clocks in each cell^2^. This enables a consolidated period of wakefulness during the day and approximately eight hours of consolidated sleep during the night. Environmental daily light exposures provide the strongest influence for the circadian system to adjust our approximately 24-hour endogenous rhythm to external clock time. Thus, light has modulating effects on our work performance, perception of wellbeing, mood and sleepiness^3^. Since we spend most of our time inside buildings, with often very low illuminance during daytime and too bright (or too blue) lighting in the evenings and night time, there is a need to better adjust and individualize lighting in humans also with regard to its biological function and influence on performance and wellbeing.

To determine circadian phase in humans, the onset of secretion of the pineal hormone melatonin in the evening is a reliable marker and can be non-invasively measured in saliva^4^. The circadian phase resetting capacities of light have been demonstrated in several well-controlled laboratory studies, depending on timing – advances after morning light, delays after evening light^5^.

There are inter-individual differences in our subjective sleep and wake timing preferences, the so-called chronotypes^6^,^7^,^8^. Extreme morning types (MT) go to bed and wake up early, whereas evening types (ET) go to bed late and wake up late, the differences may vary up to several hours. There are underlying genetic factors in these subjective preferences^9^,^10^,^11^,^12^, which may be manifested in differences in the endogenous period length of the circadian clock^13^. These differences are such that in peripheral circadian oscillators, for example clock genes in human fibroblasts^13^, and also in the dynamics of core body temperature and melatonin secretion, there is a significant association between shorter circadian periods and increasing morningness^14^. It has been suggested that extreme MT therefore need to rather to delay their endogenous rhythm on a daily basis in order to remain synchronised with the external 24-hour day-night cycle when compared to intermediate chronotypes. In ET on the other hand, who are delayed, and may have longer endogenous circadian periods, their sleep-wake rhythm needs to be advanced for them to remain entrained to the external 24-hour day^15^.

Extreme early and late chronoytpes differ also in their subjective and objective build-up of sleep need (homeostatic sleep pressure) during wakefulness^16^,^17^. Extreme MT tend to have a steeper increase of sleepiness during the day and faster dissipation of homeostatic sleep pressure during night-time sleep than extreme ET^16^,^17^,^18^. The timing of cognitive functions between extreme chronotypes differs not only by clock time, but also when tested at the same ‘internal times’^19^,^20^,^21^. Extreme ET performed better and had higher brain activation than MT in their subjective evening hours, with greater alertness in their fastest responses^20^,^21^. The authors also showed that the locus coeruleus and the area containing the suprachiasmatic nucleus were more activated in extreme ET than in MT during the evening hours^20^.

Under daily life conditions, and relative to clock time, MT get less light in the late evening (because they go to bed early) and ET get less light in the morning hours (because of their late habitual wake times)^15^,^22^,^23^. Two studies also compared natural light exposures with respect to internal circadian phase (i.e. dim light melatonin onset; DLMO) which occurred earlier in the morning for MT than in ET (i.e. at an earlier clock time^15^,^22^). It was also shown that the phase angle between circadian phase and wake-up time is longer in MT than ET, which indicates that MT wake up at a later phase of their circadian rhythm^15^,^24^,^25^. With relation to circadian phase of the DLMO, extreme MT received more light in their phase delaying portion of the day (in the evenings), whereas extreme ET received more light in their phase advancing portion in the mornings^15^,^22^. These results suggest that under naturalistic conditions, habitual light/dark cycles keep both chronotypes entrained and foster stabilization of the circadian system^15^,^22^.

To date, little is known about inter-individual differences in response to light. Taking extreme chronotypes as a ‘natural model’ of inter-individual differences in the habitual timing of sleep-wake cycles, how would such individuals behave if they had the opportunity to freely choose lighting conditions during a 16-hour day? Would they prefer lighting which reinforces their circadian entrainment, or that which enhances their daily sleep-wake preferences, e.g. delay or advance them even more? And how are diurnal variations of subjective sleepiness, mood, wellbeing and cognitive performance impacted by their choice of lighting? We aimed at testing these questions by applying two different lighting conditions together with control lighting to extreme chronotypes during an entire waking period at their preferred timing. We especially wanted to answer the question whether extreme chronotypes would choose lighting which better stabilises their entrainment to the external 24 h day.

## Results

### Habitual sleep and circadian phase angles in extreme chronotypes

Based on the screening criteria for this study (see Supplemental file) there was a significant difference between both chronotypes in the scores of two validated morning-eveningness questionnaires (the Horne Ostberg^6^ and the Munich Chronotype Questionnaire scores^7^; Table 1). The habitual bed- and wake-up times (based on the average from 7 days before the study began), midsleep on free days, sleep duration corrected (MFSc), and the dim light melatonin onset (DLMO; see below), differed between the two groups by approximately 4 hours (p < 0.0001). The habitual wake-up and bed- time for MT was at 6:19 ± 36 and at 22:13 ± 41 respectively; for ET at 10:17 ± 01:14 and at 02:10 ± 00:57, respectively (clock times; mean ± SD). In order to compare both chronotypes with respect to the timing of events, habitual wake time is referred to as circadian time 0 (=CT 0) for both chronotypes. The average study begin was scheduled one hour after habitual wake time (e.g. at CT 1; and was for MT at 7:16 ± 0:34, and for ET 11:14 ± 1:01). There was no significant difference in sleep duration or any of the circadian phase angles (=time intervals between DLMO and habitual bed and wake time as well as DLMO and mid sleep; p > 0.2), indicating that both chronotypes were tested at similar circadian phases relative to the timing of their habitual sleep-wake cycles (Table 1). From two participants (both ET), the results of cognitive, subjective and cortisol data of the last six hours of one study session had to be excluded from analyses due to medical reasons (stomach and headache).

### Self-selected lighting conditions and visual comfort

**Figure 1:**
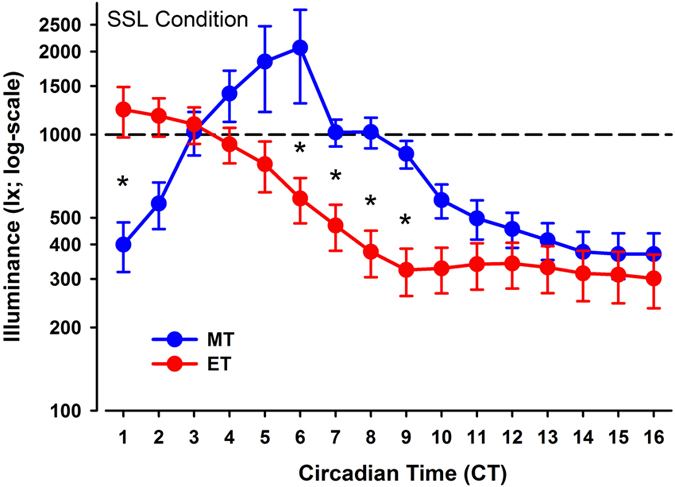
Time course of vertical illuminance (log-scale) for both chronotypes in the self-selected lighting (SSL) condition (means ± SEM). The vertical dashed line indicates the constant bright light condition (=1000 lx). Blue symbols = MT and red symbols = ET. The x-axis indicates circadian time (CT; where CT 0 = wake time); *significant difference between MT and ET (p < 0.05).

All participants had chosen available daylight as the primary light source in the self-selected lighting condition with addition of electrical lighting in the morning and evening hours (and with overcast skies). The average illuminance (measured at the vertical eye level and termed E_V_) across all participants in the self-selected lighting condition was 472 ± 5 lx, (median ± SEM), with a trend for higher E_V_ in MT (535.3 ± 74.1 lx) than in ET (408.7 ± 35.8 lx; p = 0.09; Fig. 1). There was a significant interaction with the factors ‘chronotype’ × ‘time’ × ‘lighting condition’ (with the conditions bright and self-selected lighting; P < 0.0001; F_15,930_ = 5.6). Post-hoc tests revealed higher illuminance for the self-selected lighting during the first study hour in ET than MT (p = 0.0004) and significantly higher illuminance for MT than ET from CT 6 to CT 9 (p < 0.0001). The hour with the highest illuminance occurred at an earlier circadian phase for ET than MT (CT 1 vs. CT 6). The MT chose a lighting which was significantly lower than bright light during the first two (CT 1, CT 2) and the last seven study hours of the study (CT 10–CT 16). In ET, illuminance in the self-selected lighting condition was not significantly different from bright light during the first 5 hours but was significantly lower for the remaining study (CT 6–CT 16; p < 0.05). The time course of the correlated color temperature (CCT) mirrored the time course of illuminance. ET had significantly lower CCT at CT 9 and 10 than MT (p < 0.05; see Supplemental Figure S3).

Visual comfort was evaluated as being highest in the self-selected lighting condition (p < 0.05), with some differences between MT and ET (see Supplemental file for more details).

### Time course of salivary melatonin and cortisol concentrations

For salivary melatonin concentrations the onset in dim light (DLMO) occurred at a significantly earlier clock time for MT than for ET (p < 0.0001; MT: 19:33 ± 0:56 and ET: 23:26 ± 1:48; Table 1). Despite these differences in clock times, the DLMO occurred in both chronotypes at a similar circadian phase (MT: CT 13:14 ± 00:44; ET 13:08 ± 1:19 h; p = 0.7; Fig. 1). Women had higher salivary melatonin concentrations than men in the dim condition (p = 0.0002), and therefore, hormonal analyses were performed on standardized data, (z-transformation). For melatonin, there were significant interactions with the factors ‘time’ × ‘condition’ (F_30,1342_ = 23.46; p < 0.0001) and ‘condition’ × ‘chronotype’ (F_2,1342_ = 6.14; p = 0.002) and a significant interaction ‘time’ and ‘chronotype’ (F_15,1342_ = 1.76; p = 0.036), but the three-way interaction with all factors was not significant (p = 0.96).

**Figure 2:**
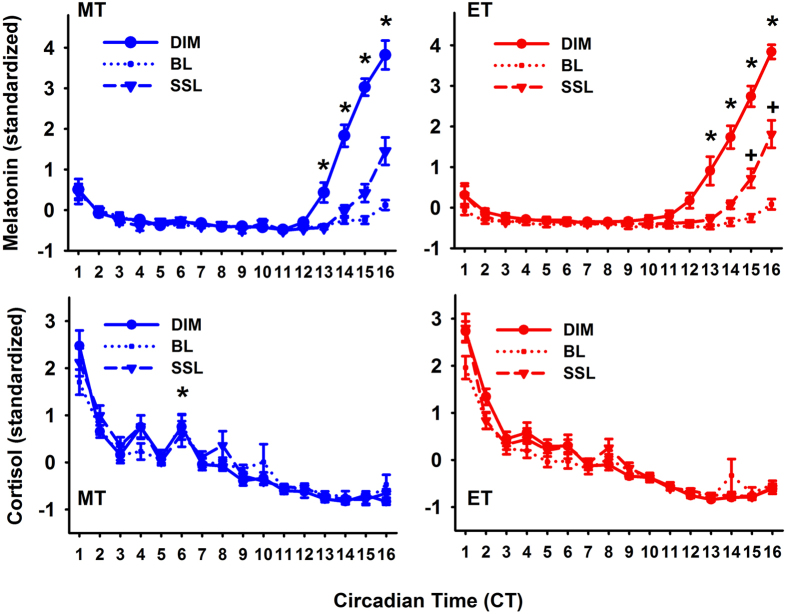
Time course of salivary melatonin concentrations (upper graphs) for morning types (MT; blue symbols; left) and evening types (ET; red symbols; right) for all three lighting conditions (dim light = DIM; constant bright light = BL; self-selected lighting = SSL; N = 32; means ± SEM). *Differences between DIM and both SSL and BL (p < 0.05); ^#^significant difference for SSL and BL conditions (‘condition’ × ‘time’ for MT and ET separately p < 0.05). The data was standardized (z-transformation) and is plotted relative to habitual wake time (=CT 0). The lower two graphs show the time courses of salivary cortisol concentrations, again for both chronotypes: morning types (blue symbols; left) and evening types (red symbols; right) on standardized data. *Indicate significant differences between morning and evening types (main effect of ‘chronotype’; p < 0.05; see result section for exact p- and F-values).

In a next step we tested whether the time course of melatonin between all lighting conditions was different within each chronotype (Fig. 2, upper graphs). For MT, there was a significant difference between dim and bright light as well as between dim and self-selected lighting between CT 13–16 (without a significant difference between bright and self-selected lighting at any time point; ‘condition’ × ‘time’; F_29,666_ = 13.8; p < 0.0001). For ET, melatonin was lower in bright than dim light between CT 12–CT 16, and it was also lower in the self-selected, when compared to dim light between CT 13–16. In addition, there was a significantly higher melatonin concentration during self-selected lighting in ET when compared to bright light between CT 15 and 16 (condition × time; F_29,676_ = 11.04; p < 0.0001). Taken together, these results indicate a similar time course for the dim and bright light condition in both chronotypes, but a difference in the dynamics during the self-selected lighting when compared to bright light in ET, but not MT (Fig. 2; upper graphs).

In dim light, salivary cortisol concentrations were higher in MT than ET (main effect of ‘chronotype’; F_1,29_ = 8.9; p = 0.006), but there were no significant sex differences (p = 0.19). The data was then also standardized and with all three lighting conditions, there was a significant interaction with the factors ‘condition’ × ‘time’ (p = 0.049, F_30,888_ = 1.48) and ‘chronotype’ and ‘time’ (p = 0.006, F_15,450_ = 2.22), such that in MT, cortisol concentrations were significantly higher at CT 6 than in ET (Fig. 2; lower graphs). Next we wanted to specifically determine the time course and differences between the three lighting conditions within each chronotype. There was a significant variation over time for MT and ET (when they were tested separately), with a significant decrease until CT 3 for both chronotypes and for MT again an increase in cortisol concentration at CT 6 (main effect of ‘time’; F_15,225_ > 78.7; p < 0.0001). There were no differences between the three conditions or interaction between ‘condition’ and ‘time’, neither in MT or ET (p > 0.18).

### Wellbeing, subjective alertness, mood and mental effort

**Figure 3:**
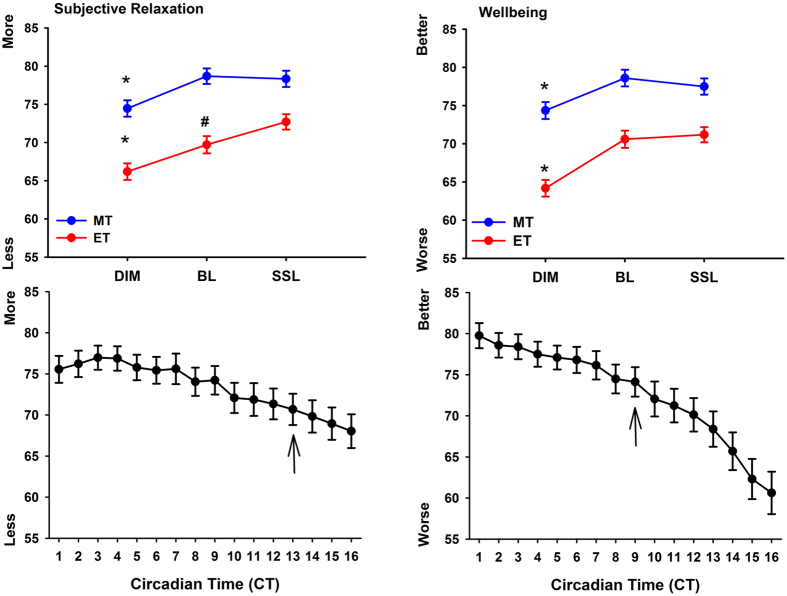
Upper two graphs: averaged values for subjective relaxation (left side) and wellbeing (right side) per light condition (DIM; BL; SSL) for both chronotypes (MT = blue symbols; ET = red symbols; means ± SEM). *Significant differences between DIM - BL and DIM and SSL within each chronotype (p < 0.05); ^#^significant differences between BL and SSL in ET only (p < 0.05; ‘condition’ × ‘chronotype’). The lower two graphs show the time course for subjective relaxation (left side) and wellbeing (right side) across all three lighting conditions (main effect of ‘time’). The black arrows indicate the beginning of the progressive worsening, when compared to CT 1 for both chronotypes (main effect of ‘time’; p < 0.05).

The ratings of subjective relaxation showed that both chronotypes felt more tense in dim light when compared to bright and self-selected lighting, whereas ET felt also more relaxed in self-selected than in bright light (‘condition’ × ‘chronotype’; F_2,1397_ = 4.11; p = 0.02; Fig. 3 upper graphs; left). Participants became more tense in general after CT 13 (main effect of ‘time’; F_15,1397_ = 8.57; p < 0.0001; Fig. 3 lower graph; left). Physical wellbeing was significantly higher in bright and self-selected lighting when compared to dim light in both chronotypes (main effect of ‘condition’ (F_2,1396_ = 33.9; p < 0.0001; Fig. 3 upper graphs right) and became worse toward the end of the study session (i.e. after CT 9; main effect of ‘time’; F_15,1396_ = 22.9; p < 0.0001; Fig. 3; lower graph right).

**Figure 4:**
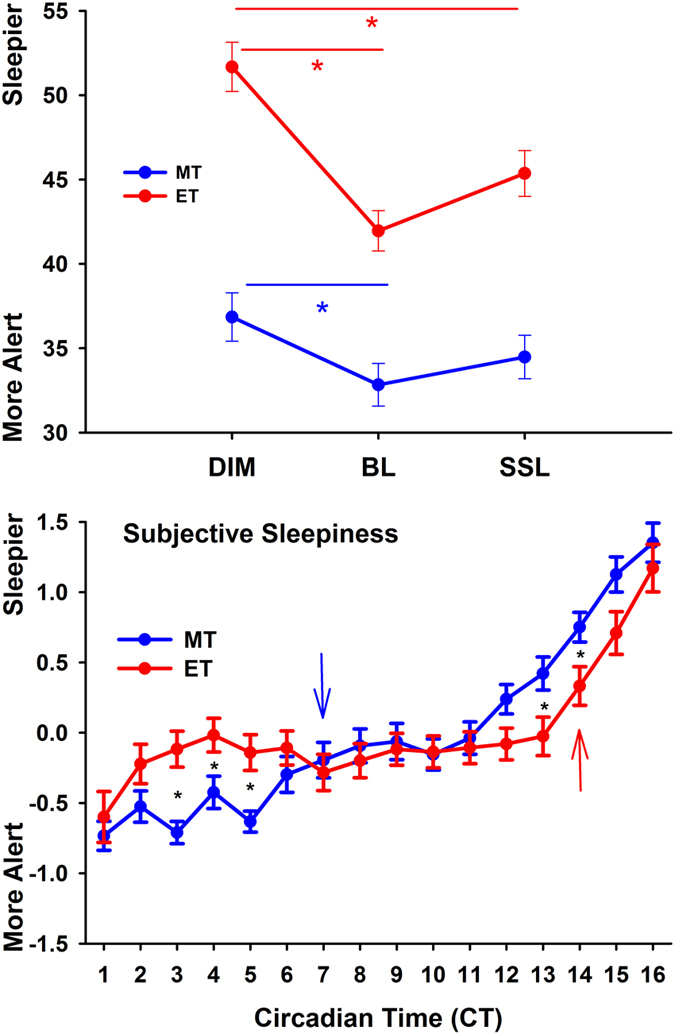
Subjective sleepiness per condition and chronotype (upper graph; blue symbols = MT; red symbols = ET); *significant differences within each chronotype and condition (N = 32; means ± SEM; ‘chronotype’ × ‘condition’; p < 0.05). The lower graphs shows the time course of subjective sleepiness (standardized data) for MT (blue symbols) and ET (red symbols). The blue and red arrow show the increase in sleepiness since CT 1 (=one hour after habitual wake time) for the respective chronotypes.

The ET felt significantly sleepier than MT overall (absolute values; main effect of ‘chronotype’; F_1,29_ = 5.1; p = 0.032) and both chronotypes were more alert in the bright light than the dim light conditions, whereas only ET were also more alert in self-selected lighting than dim light conditions (‘chronotype’ × ‘condition’; F_2,1397_ = 3.22; p = 0.04; Fig. 4 upper graph). There was also a significant interaction with the factors ‘chronotype’ and ‘time’ (F_15,1397_ = 4.1; p < 0.0001). Post-hoc tests for this interaction with standardized data (Fig. 4; lower graph) showed that ET were sleepier than MT between CT 3 and CT 5 and MT were sleepier than ET from CT 13 to CT 14 (p < 0.012). When compared with CT 1, MT became significantly sleepier after CT 7 and ET at CT 4 and again after CT 14 until the end of the study (p < 0.045).

Mood ratings were significantly worse for both chronotypes under the dim than both, the bright light and self-selected lighting conditions (main effect of ‘condition’; F_2,1397_ = 10.66; p < 0.0001) and became also worse after CT 14, when compared to the beginning of the study at CT 1 (main effect of time; F_15,1397_ = 3.7; p < 0.0001). There was no difference between the bright light and the self-selected lighting condition or significant variation in the time course between chronotypes (p > 0.35; data not shown).

In the mental effort scale, men put more effort in the cognitive tasks overall (p = 0.008) and mental effort was higher in dim light than self-selected and bright light (main effect of ‘condition’; F_2,1397_ = 47.56; p < 0.0001). The dynamics across all conditions showed that mental effort was greater for CT 6, CT 8 and CT 10 and then again for CT 12–CT 16, when compared to the beginning of the study F_15,1397_ = 7.82; p < 0.0001; main effect of ‘time’). The variations of mental effort, especially in the first half of the study protocol are most likely reflecting differences between the mental loads for the two cognitive test batteries, such that mental load was higher with the version of the visual n-back and the Go-no-go test (see Supplemental Figure S4).

### Cognitive performance

**Figure 5:**
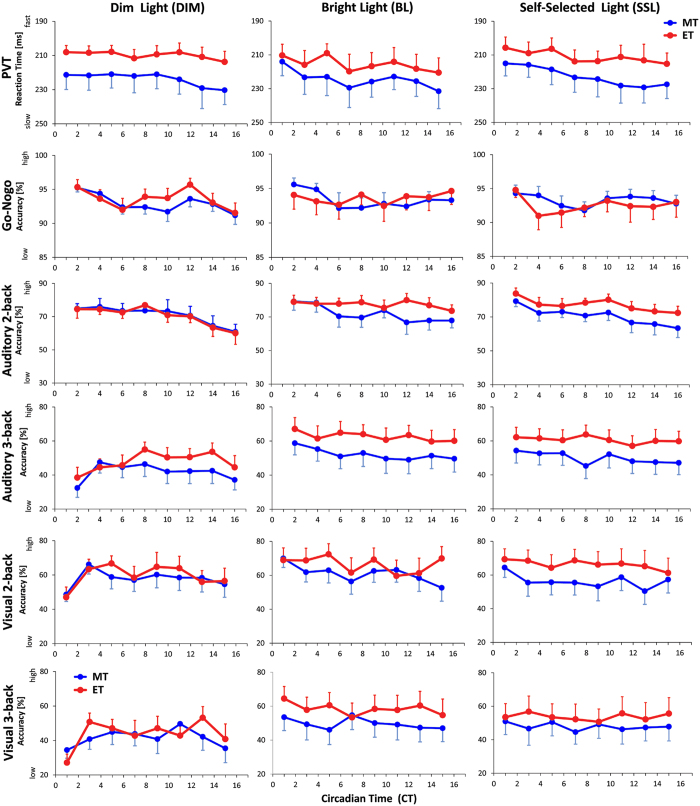
Time course for reaction time in the PVT and accuracy of cognitive performance tests (Go-no-go; auditory 2-, and 3-back; visual 2- and 3-back) for both chronotypes (morning types = blue symbols; evening types = red symbols). DIM = left column; BL = middle column; SSL = right column; N = 32; means; SEM).

In the sustained attention tasks there was overall highest accuracy for the ‘Go-no-go’ at the beginning with a significant decline between CT 6 and CT 10; and again between CT 14 and CT 16 (main effect of ‘time’; F_7,684_ > 4.49; p < 0.0001). For the PVT, reaction times became significantly slower towards the end of the study (main effect of ‘time’; F_7,684_ = 5.25; p < 0.0001), without significant differences between lighting conditions or chronotype in both sustained attention tasks (Fig. 5).

In the n-back tasks assessing higher cognitive performance (working memory), ET performed significantly better in the auditory 2-back task in bright and in self-selected lighting than in dim light; without differences for MT between the three lighting conditions (F_2,684_ = 6.76; p = 0.001; ‘condition × chronotype’). In the visual 2-back task, ET again performed better in bright light than in dim light (‘condition’ × ‘chronotype’; F_2,684_ = 3.01; p < 0.05; Fig. 5), without a significant difference between dim light and self-selected light (p = 0.06). There was a variation over time for both chronotypes in the auditory 2-back, with significantly lower accuracy in the last three sessions (i.e. after CT 12; main effect of ‘time’; F_7,684_ = 11.49; p < 0.0001). For both 3-back versions, accuracy was significantly worse in dim than in bright and self-selected lighting for both chronotypes (main effect of ‘condition’; F_2,684_ > 33.0; p < 0.0001). Both versions of the 3-back task were significantly more difficult than the 2-back (F_1,150_ > 58; p < 0.0001; main effect of n-back version) which was also reflected in slower RTs in both 3-back versions (p < 0.0001). The auditory 0-back served as control and accuracy declined over time (from 89.7% at CT 2 to 81.0% at CT 16; p < 0.0001; data not shown), however, there were no significant differences between lighting conditions and chronotypes, mirroring the pattern in the sustained attention tasks.

## Discussion

We investigated whether extreme chronotypes would choose differential lighting during a habitual waking period of 16 hours. A self-selected lighting condition was compared with constant bright light and a control in dim light to determine whether the self-selected lighting environment would differentially affect chronotypes’ hormonal secretion of melatonin and cortisol, as well as subjective sleepiness, wellbeing, mood, visual comfort and cognitive performance.

We found that all participants took advantage of available natural light (without direct window view) and chose it as the main light source during daytime. The diurnal distribution of illuminance and the color temperature of light however, was different between both chronotypes such that MT received more light in the late afternoon and early evening hours, whereas ET received more light in their morning hours.

We found higher melatonin concentrations in women than men which corroborate results from the literature^26^,^27^,^28^. However, the reasons for these sex differences are not well understood. Oral contraceptives, lighting, physical activity and menstrual phase may play a role. Since we controlled for lighting and physical activity, it might be that menstrual phase and oral contraceptives at least partly had accounted for the sex differences in melatonin concentrations. We could not confirm longer circadian phase angles as shown by others^15^,^24^ and therefore, both chronotypes of our study were tested at similar circadian phases. In consequence of this, melatonin onsets in dim light (DLMO) occurred at similar circadian times for both groups, and this was significantly earlier when compared to the onsets in bright and self-selected lighting. Only in the self-selected lighting was the melatonin onset also significantly earlier in ET but not in MT, when compared to bright light. Since illuminance was similar in both chronotypes after CT 9, these differences in the melatonin onset in the self-selected lighting condition could therefore solely be explained by different illuminance and/or higher color temperatures (i.e. blue light) in the hours before. The differences were such that MT received significantly higher illuminance from CT 6–9 and higher color temperatures than ET between CT 9 and CT 10. Compared to the phase response curve for bright light (PRC^5^), this would be in the phase delaying portion; whereas higher illuminance in ET (than in MT) occurred at CT 1, which is in the phase advancing portion of the PRC. Since bright light of 1000 lx (and higher) is suggested to have saturation effects for circadian phase shifts (as shown in the laboratory during nighttime)^29^, illuminance levels in ET were in favour for circadian phase advances. On the other hand, MT spent more time in the delaying portion of the PRC (i.e. from CT 6 to CT 9) when compared to ET and received higher color temperatures (i.e. greater proportions of short wavelength, i.e. blue light). Both chronotypes had therefore chosen lighting conditions which rather stabilized their circadian phase as has been already demonstrated under habitual daily light exposures^15^,^22^.

Despite working on time schedules which were adjusted to the participants’ preferred waking hours, MT and ET performed differently in some of the tests during both the constant bright and the self-selected lighting conditions when compared to dim light. Whereas MT showed no differences between lighting conditions in the 2-back tests, the ET performed better in the bright (auditory version and visual version) and the self-selected (auditory version) lighting condition as compared to the dim light. MT also reported greater mental effort, especially with certain tasks (visual n-back, Go-no-go test), reflected in higher cortisol secretion than ET during all study conditions at CT 6. Although there is evidence that MT show a higher cortisol awakening response^30^, we could not determine this here, because the study protocol started one hour after wake time. However, the salivary cortisol levels between chronotypes did not differ in the first hour of the study, but were overall higher in MT than ET, most likely due to higher efforts in their subjective morning hours and after the more demanding cognitive test batteries (taken every two hours).

The ET expressed a higher level of subjective sleepiness than MT throughout the study, which is consistent with earlier work^31^. Higher subjective but not objective sleepiness (as assessed during the wake maintenance test) was found in ET than MT, when tested during an extended period of 36 hrs of wakefulness^31^. Since we did not assess objective sleepiness (for example by electroencephalography; EEG) we cannot exclude that objectively, our ET were objectively not sleepier than MT. However, the fact that ET performed equally well as MT in the cognitive tests, especially in the sustained attention tests despite greater subjective sleepiness suggests that ET might not have been sleepier than MT. On the other hand, the relative dynamics of subjective sleepiness across 16 hours showed an earlier increase of subjective sleepiness in the second half of the waking period in MT than ET (Fig. 4), and thus, corroborates the faster build-up of homeostatic sleep pressure in MT, as shown by others^17^,^18^. There is also evidence that ET can better cope with higher subjective sleepiness than MT (as suggested for example by Taillard *et al*.^31^). The earlier increase of subjective sleepiness (see above) could have a more detrimental influence on cognitive performance in MT than ET. Furthermore, it has been suggested that one of the key mechanisms for behavioural differences between extreme chronotypes might be the stronger negative input from sleep homeostasis on the anterior hypothalamic region which is involved in circadian rhythm generation^20^, which might explain some of the differences between lighting conditions within both chronotypes in the working memory test.

It is important to emphasise that all our participants were tested during their subjective wake time, aligned according to their preferred habitual circadian phase and with respect to their endogenous sleep/wake rhythm. We thus compared the participants at their same endogenous circadian phases. It will be important to study extreme chronotypes at the same local clock time, for example during ‘normal’ office hours (i.e. 8:00–17:00), which would most likely lead to different results because of misalignment problems (mostly in ET) and so-called ‘social jet lag effects’^32^. Such a study would also challenge the choice of lighting conditions, especially if extreme chronotypes share working spaces, as suggested by Begeman^33^. In the same vein, it would also be crucial to further determine, whether extreme chronotypes differ also in their subjective and objective light responses due to underlying different reactivity to light as recently was suggested^34^.

Taken together, although there were significant differences between the constant bright and the self-selected lighting conditions, the differences between chronotypes were rather small. Because constant bright light exposure in the evening is known to induce a strong circadian phase delay, self-selection of lighting would be the better option when considering that adequate lighting is required for high performance at working places. The self-selected lighting has also the advantage to be more energy efficient, as we recently reported such that approximately 40% of the energy demand used for the bright lighting can be saved^35^. Thus self-selected individualised lighting offers a practicable solution in office spaces to positively modulate biological functions, wellbeing and visual comfort, especially if work schedules are flexible.

## Methods

### Study Design

All participants underwent three different lighting conditions (three different sessions) in a within-subject design across all seasons; each session lasted for 16 hours during habitual wake hours under constant posture conditions. The first session was always in dim light and served as control condition. The two experimental lighting conditions were constant bright light (1000 lx) and self-selected lighting. These two conditions were scheduled in a balanced-cross over design. All three sessions took place within a time frame of 3 to approximately 6 days.

### Participants

Thirty-two participants [16 extreme morning types (MT); 16 extreme evening types (ET); 14 men and 18 women; mean age 22.7 ± 3.5 years; (±SD), age range 18–31 years; see Table 1] completed all 3 sessions of the study. The study procedures were approved by the local Ethical Review Board in Lausanne (Switzerland) and were in agreement with the tenets of the Declaration of Helsinki. All participants provided written informed consent.

### Study Protocol

Seven days before each of the study sessions subjects were asked to maintain a regular sleep-wake rhythm–with approximately 8 hours of sleep, within ±30 min of self-selected target times, based on their habitually chosen bed times. Compliance was controlled by a wrist worn activity monitor (Daqtix^®^, Oetzen-Süttorf, Germany) and sleep logs. On their way to the laboratory in the morning, participants wore dark goggles to avoid any bright light exposure before the study sessions.

Each of the study sessions (see Supplemental Figure S1) was scheduled to begin one hour after the assigned habitual subjective wake-time [=circadian time 1 (CT 1)] and lasted for 16 hours (end of CT 16). During the study sessions, participants remained seated in the testing room. They were allowed to read, perform paper work or listen to music (including one hour of scheduled computer work in the middle of the day). The study protocol was identical for all three sessions. Every 30 minutes, participants were asked to assess their subjective wellbeing, mood and sleepiness on visual analogue scales (VAS; see Supplemental file). Cognitive tests were performed every hour with two alternating test batteries (each was given 8 times per day). We used an auditory and a visual n-back test, two sustained attention tests [the Psychomotor Vigilance Test (PVT) and the Go-no-go test; see also the Supplemental file]. Immediately after each cognitive test battery, participants were asked to evaluate their mental effort they needed to perform the tests on the Mental Effort Rating Scale (MERS^36^; see Supplemental file). Approximately 15 minutes after the end of each cognitive test battery, a saliva sample for hormonal analyses was taken.

The dim light condition (=low light intensity control condition) had an E_V_ of less than 5 lx with use of indirect electrical light only. The constant bright light condition E_V_ was targeted to be constant at 1000 lx. We used daylight, and at times of insufficient daylight, ceiling lights with polychromatic white light (4000 K) were switched on to provide the target illuminance. The constant E_V_ of 1000 lx was chosen based on the literature^37^,^38^ where light exposures had an effect, and it was aimed to provide a highly saturated condition for non-visual functions (=high light intensity control condition). During the self-selected lighting condition, participants could choose daylight and/or electrical light from all available light sources (see Supplemental file for detailed information), the maximum E_V_ provided by electrical lighting was approximately 1200 lx. Illuminance and color temperature were dependent on the subject’s choice. Participants were asked to assess their lighting preference every 60 min, which was then adapted accordingly. Visual acuity and contrast was assessed every two hours and visual comfort was assessed every 30 min by a visual analogue scale (see Supplemental file).

Salivary samples for analyses of hormonal concentrations were collected every hour, 15 minutes after completion of the cognitive tests. Based on the time of the rise of melatonin concentrations in the evening dim light during the first session, we assessed the dim light melatonin onset (DMLO). The DLMO was defined as the time when salivary melatonin concentrations crossed the threshold of the lowest 3 daytime value plus 2 SD and was calculated by linear interpolation by using the software tool validated by Danilenko *et al*.^39^. Several circadian phase angles were calculated to indicate the time interval between the habitual DLMO and bedtime, wake time and midsleep (based on the MSF-Sc). Visual acuity and contrast was assessed every two hours and visual comfort was assessed every 30 min by a visual analogue scale (see Supplemental file).

## Additional Information

**How to cite this article**: Maierova, L. *et al*. Diurnal variations of hormonal secretion, alertness and cognition in extreme chronotypes under different lighting conditions. *Sci. Rep*. **6**, 33591; doi: 10.1038/srep33591 (2016).

## Supplementary Material

Supplementary Information

## Figures and Tables

**Table 1:** Demographics and averaged habitual wake- and bedtimes, study begin and end times (mean ± SD) of extreme morning types (MT; N = 16) and extreme evening types (ET; N = 16).

Variables (mean ± SD)	Morning Types (MT) (N = 16)		Evening Types (ET) (N = 16)
Sex (M/F)	7/9	ns	7/9
Age (years)	22.0 ± 3.8	ns	23.3 ± 3.1
MEQ	70.5 ± 3.1	*	30.2 ± 5.0
MCTQ	2.88 ± 0.81	*	6.53 ± 0.82
PSQI	2.94 ± 1.1	ns	3.50 ± 1.9
ESS	5.38 ± 2.9	ns	5.56 ± 2.1
Waketime (hh:mm)	06:19 ± 0:36	*	10:17 ± 1:14
Bedtime	22:13 ± 0:41	*	02:10 ± 0:57
Midsleep on free days (sleep duration corrected) (MFS-Sc)	02:16 ± 0:35	*	06:15 ± 1:02
Study begin	07:16 ± 0:34	*	11:14 ± 1:01
Sleep duration	08:06 ± 0:31	ns	08:08 ± 0:40
DLMO Clock Hour	19:33 ± 0:56	*	23:26 ± 1:48
Circadian Phase Angle: BT-DLMO	02:40 ± 0:44	ns	02:44 ± 1:19
Circadian Phase Angle: DLMO-WT	13:14 ± 0:44	ns	13:08 ± 1:19
Circadian Phase Angle MSF-Sc-DLMO	06:43 ± 0:41	ns	06:49 ± 1:15

MEQ = Morningness-Eveningness Questionnaire; MCTQ = Munich Chronotype Questionnaire; PSQI = Pittsburgh Sleep Quality Index; ESS = Epworth Sleepiness Scale. WT = averaged wake time; BT = averaged bed time; DLMO = dim light melatonin onset; MSF-Sc = time of mid-sleep on free days, sleep duration corrected. The averaged circadian phase angles per chronotype are shown as the time interval between bed time, wake time, midsleep on free days, sleep duration corrected (MSF-Sc) and the DLMO (in hh:min). *Significant differences between both chronotypes (p < 0.0001); ns = not significant (p > 0.2).
