# Supplementary material for: Diurnal variations of hormonal secretion, alertness and cognition in extreme chronotypes under different lighting conditions
Source: Sci Rep. 2016 Sep 20;6:33591. doi: 10.1038/srep33591 (PMC5028769; doi:10.1038/srep33591)
Supplement: Supplementary Information [file srep33591-s1.pdf]

Supplemental File

Diurnal variations of hormonal secretion, alertness and cognition in extreme chronotypes under different lighting conditions

Maierova L[1,2], Borisuit A[1], Scartezzini J-L[1], Jaeggi SM[3], Schmidt C[4], Münch M[1,5*]

[1] Ecole Polytechnique Fédérale de Lausanne, Solar Energy and Building Physics Laboratory, CH-1015 Lausanne Switzerland; [2] Czech Technical University in Prague, UCEEB, Trinecka 1024, 273 43 Bustehrad, Czech Republic [3] School of Education, University of California, Irvine; USA; [4] GIGA-CRC in Vivo Imaging, University of Liège, Belgium; [5] Current address: Charité Universitätsmedizin Berlin, Sleep Research & Clinical Chronobiology, Institute of Physiology, Berlin, Germany, St. Hedwig Hospital, Grosse Hamburger-Strasse 5-11; D-10115 Berlin

Screening of participants

Participants were recruited via Flyers at local universities and schools. Extreme chronotypes were chosen by an interview, an entry questionnaire and two validated questionnaires to search for extreme chronotypes: the Morningness-Eveningness Questionnaire (MEQ)[1] and the Munich Chronotype Questionnaire (MCTQ)[2]. Extreme morning types (MT) had to have scores in the MEQ higher than 70 and extreme evening types (ET) a score lower than 30. For evaluation of extreme chronotypes with the MCTQ we calculated the 'time of mid-sleep on free days; sleep duration corrected'[2] (MSF-Sc). Scores greater than 5.99 were considered as extreme evening types and scores lower than 3 as extreme morning types. We chose participants with at least one of the two scores to be extreme, while the second one had to be at least moderate (MEQ or MCTQ).

Only healthy participants, non-smokers, without any medications (except for oral contraceptives) were included. Women were scheduled for the study during the luteal phase of their menstrual cycle. Shift work, transmeridian travels across more than 2 time zones within the last three months, as well as excessive alcohol or caffeine consumption, or recent drug abuse, were exclusion criteria for study participation. Good sleep quality was assessed with the Pittsburgh Sleep Quality Index (PSQI)[3] with a score ≤ 5. Due to the known very late (or very early) habitual bed-and wake-times in extreme chronotypes, often resulting in difficulties to stay entrained with normal work and social duties[4], we also included suitable participants with a maximum of 6 in the PSQI score (which occurred in 4 participants, all in extreme evening types). For the assessment of (excessive) daytime sleepiness we used the Epworth Sleepiness Scale (ESS)[5]. Scores of 10 and lower are assumed to be normal and only participants with these scores were included in our study.

Our target sample size was 32 participants (half extreme morning and half extreme evening types; men and women). Out of more than 1100 persons, who responded to our study announcements, we included 37 participants in total, based on their extreme diurnal sleep-wake preferences (see above). Three participants had to be excluded early in the study because they were not able to follow the study protocol, and two participants resigned from the study after inclusion.

Study room and equipment

The study was performed from 2011-2013 across all seasons in the testing room of the Solar Energy and Building Physics Laboratory (LESO-PB), at the Swiss Federal Institute of Technology in Lausanne, Switzerland (geographical latitude N 46.5°). The testing room is equipped with an anidolic facade system[6] which increases the daylight flux deep into the room. The windows of the study room were south oriented. Two horizontal lines of windows, each of them equipped with opaque blinds allowed control of the influx of daylight. The lower blinds were always closed in order to prevent direct outside view during the study, since it is known that direct view has an impact on the choice of daylight. Daylight entered the room only via the upper windows. Next to the external shadings, internal Californian blinds and curtains for total blocking of the exterior light were installed.

Lighting conditions

Illuminance on a vertical plane at the approximate corneal level of the participant (E_V) was continuously recorded in 5 min intervals throughout the study by using a spectroradiometer (Specbos 1201, JETI, Jena, Germany), placed on a tripod next to the subject. At times of insufficient daylight available (during the constant bright light condition) or according to the

choice of the subject (during the self-selected lighting condition), electrical lighting was used. The available sources of electrical lighting were uniformly distributed polychromatic ceiling mounted fluorescent tubes (4000 K, E_V 1280 lx, dimmable), one indirect fluorescent standing lamp (3000 K, E_V 50 lx, dimmable) and a desk lamp with an incandescent bulb (2700 K). The possible illuminance from electrical lighting ranged from 5 to 1400 lx (Ev; see also spectral power distributions of the light sources Supplemental Figure 1).

For the self-selected lighting condition, participants were asked every 60 min to adjust illuminance/brightness if needed (which was done accordingly by an assistant), or whenever they asked for a change in between these regular assessments. At the beginning of the study, all available light sources were explained and demonstrated to the participants, including shading and blinds, and electrical light sources where shown in full range (e.g. from minimum to maximum).

Visual Acuity

All participants had normal or corrected vision (contact lenses or glasses). In order to assess whether glare or visual discomfort had an impact on visual abilities, contrast and visual acuity responses were tested every second hour with the computerized 'Freiburg Vision Test' (Fract;[7]). Thereby, participants had to indicate the direction of the presented so called ‘Landolt-Ring’ (which consists of different shapes and orientations of a 'C') on the desktop screen, by pressing the correct arrow key on the keyboard. The size of the Landolt rings and/or the contrast changed during the test. There were no differences between the bright and the self-selected lighting condition, but in the control condition in dim light, contrast discrimination was significantly lower for both chronotypes ($p<0.0001$). The reason for this difference was the brightness of the computer screen, which was lowered in the dim light condition in order to keep

illuminance below 5 lx. All participants had normal acuity (tested in the Fract[7]). When we tested the reaction times for acuity in the Fract between the bright and the self-selected lighting, MT responded faster in the self-selected lighting than the bright light condition ($p=0.0002$; ‘condition’ x ‘chronotypes’; $F_{1,144}=14.0$) but with equal contrast responses ($p>0.3$). Otherwise, there was no effect of time or an interaction between the factors 'time', 'condition', 'chronotype' or an impact by the covariate 'sex' ($p>0.2$).

Visual comfort

For assessment of visual comfort, we used 11 items out of the Office Lighting Survey[8] and we used the items on a visual analogue scale. For all items there was a significant interaction with the factors ‘chronotype’ and ‘condition’; ($F>4.1$; $p<0.007$; see Supplemental Table S1). The single factors 'time' or 'chronotype' were not significant for none of the items. However, some of them were not normally distributed after log-transformation and therefore, all post-hoc analyses were performed with the non-parametric Kruskal-Wallis Test (on absolute data).

Both chronotypes had highest visual comfort in the self-selected lighting (SSL) condition (item 1, item 2) and perceived that there is not enough light to work/read properly only in dim light (DL; item 3). The latter was significantly more pronounced in ET than MT. For the differences between the DL and the bright lighting (BL), only ET found that there was rather too much light to properly read/work in BL than DL (item 4) and they perceived significantly more glare in BL and SSL than in DL (item 6). For both chronotypes, the light distribution was worse in the DL than the other two lighting conditions (item 5), without a significant difference between BL and SSL. Both chronotypes found that their skin did rather not look more unnatural in any of the three lighting conditions (item 7). For item 8, only MT evaluated the DL condition as rather not too 'warm' and MT and ET found that the SSL was not too cold when compared to DL (item

9). The DL condition was worse than BL and SSL for both chronotypes, when they compared it to their usual working conditions and they would spend significantly fewer hours in DL than in BL and SSL (item 10 and 11).

Visual analogue scales for subjective evaluations of wellbeing, sleepiness, mood and mental effort

Participants had to indicate their subjective sleepiness, wellbeing and mood every 30 minutes on a paper based version by means of a pen on a vertical line of 100 mm between two extremes (for example for sleepiness: 0 mm= extremely alert; 100 mm= extremely sleepy; mood: 0 mm=very good mood; 100mm=very bad mood). For graphical illustration, the mean values for Figure 3 (subjective relaxation and wellbeing) were subtracted from 100. After each cognitive testing participants were asked to evaluate the mental effort required to perform the cognitive test battery on the paper-based Mental Effort Scale[9] (see also Supplemental Figure S4).

Cognitive Testing

Each of the cognitive test series contained either a visual or auditory version of the n-back test, which assesses higher cognitive functions such as working memory[10,11]. Three difficulty levels were used for the auditory n-back (0-, 2- and 3-back) and two levels in the visual n-back (2- and 3-back). The auditory n-back trial consisted of 5 blocks of the 0-, 2- and 3-back version. Each trial consisted of 30 stimuli with 9 different French spoken letters. The stimuli order was randomized for each trial with fixed ratios of correct vs. incorrect answers (1:3)[10]. In the visual n-back version, 3 x 2 blocks of both n-back versions (2- and 3-back) with randomized asymmetrical abstract forms as yellow stimuli (black background) were presented on the computer screen[12]. Again, the correct-to-false ratio was 1:3. In both, the auditory and visual versions of the task, the

participants had to indicate whether the currently presented stimulus is identical to the one presented two (=2-back) or three (=3-back) steps before by pressing a button for 'yes' or 'no' on the keyboard. For the 0-back (control task), participants had to react when a ‘K’ was presented by pressing a button on the keyboard (only used in the auditory n-back version). Accuracy (correct answers minus false alarms), and response time for correct answers were calculated for all n-back tests. Every hour, either the auditory or the visual n-back versions were alternately presented.

Sustained attention was assessed with the auditory version of the Psychomotor Vigilance Task (PVT) [13] which lasted for 10 minutes and was carried out at 2 hour intervals (i.e. 8 times) during each study condition. Median reaction times (RT) were recorded as the main outcome variable. Any anticipatory responses (RT <100 ms) and lapses (RT >500 ms) were removed from the data prior to analyses. In the alternating hours, a Go-no-go test was performed, assessing cortical inhibition, sustained attention and response control. During this task, two different letters (‘M’ and ‘W’) were presented on the screen in a continuous, randomized order. Participants were required to press ‘y’ when the letter ‘M’ was presented and not to press, when a ‘W’ was presented. Accuracy and reaction times were measured for correct trials.

Salivary Samples

After the study session, samples were centrifuged and frozen at -20°C. Upon completion of the study, all samples were sent to an external laboratory for radio-immuno assays (RIA) of melatonin and cortisol (Dr. B. Middleton, University of Surrey, Guildford GB). The inter-assay coefficient of variability (CV) for low and high melatonin was 12.4% and 8.5% respectively; and for low and high cortisol: 12.6% and 10.7%. The intra-assay CV for low and high melatonin was 6.9% and 2.4%; and for low and high cortisol: 10.8% and 5.3%. The limit of detection was 0.4 nmol/l for cortisol and 0.6pg/ml for melatonin.

Statistics

All variables were averaged per hour except for the cognitive tests (which occurred every 2 hours). Data for Table 1 (demographics and habitual sleep-wake times) were analyzed by applying two-sided t-tests. Missing data points in the cognitive tests (due to technical problems) were linearly interpolated (i.e. for the auditory 2- and 3-back in 1.2% and 3% of the tests, respectively). The software SAS (SAS Institute Inc., Cary, NC, USA; v9.3) and Statistica (v9.1 StatSoft, Inc. Tulsa; USA) was used for statistical analyses. Data which did not meet the criteria for a normal distribution (i.e. Kolmogorow-Smirnow test $p<0.05$) were transformed [$\log_{10}$ or square root; for melatonin, subjective relaxation: $\log_{10}$ (value + 1)]. For analyses of the auditory 0-back test and the visual comfort, we used non-parametric tests (Friedman-Anova, Kruskal-Wallis test) since the data did not meet criteria for a normal distribution even after transformation. The analyses with hormone concentrations (melatonin, cortisol) as well as subjective sleepiness recordings were done on log-transformed and standardized data (by applying a z-transformation). Linear mixed models (PROC MIXED) were used for analyses with the repeated factor 'time' (CT1-16), the fixed factors 'condition' (DIM, BL, SSL) and 'chronotype' (MT, ET). The factor 'sex' (female, men) was added as covariate. Degrees of freedom were approximated after Kenward Rogers. For post-hoc analysis, the Tukey-Kramer test (with adjusted *p*-values for multiple comparisons) was applied.

Supplemental Table S1

Item	Chronotype	MT			ET		
	**Assessment**	**DIM**	**BL**	**SSL**	**DIM**	**BL**	**SSL**
1	'I like the light in this room' (yes=0; no=100)	53.3 (29.7)	27.5^{a} (25.9)	9.1^{b,c} (11.1)	61.3 (31.8)	23.4^{a} (23.2)	10.0^{b,c} (11.4)
2	'Overall, the lighting in this room is comfortable' (yes=0; no=100)	51.8 (28.7)	27.7^{a} (25.1)	8.5^{b,c,} (11.6)	59.7 (31.6)	24.3^{a} (23.3)	9.7^{b,c} (10.9)
3	There is not enough light to work/read properly' (yes=0; no=100)	46.6^{d} (35.8)	88.4^{a} (22.0)	87.9^{b} (24.2)	24.0 (26.4)	93.2^{a} (12.5)	92.4^{b} (9.9)
4	'There is too much light to work/read properly' (yes=0; no=100)	90.0^{d} (11.2)	81.5 (23.4)	92.8^{c} (11.5)	94.9 (6.6)	72.7^{a} (28.2)	89.9^{c} (20.3)
5	'The light in this room is not well distributed' (yes=0; no=100)	54.4 (29.3)	86.7^{a} (19.9)	80.1^{b} (26.7)	45.4 (29.8)	89.0^{a} (17.0)	85.0^{b} (21.8)
6	'How much glare do you perceive in this room' (not perceivable=0;intolerable=100)	25.1^{d} (21.1)	32.3 (20.6)	22.5 (17.8)	7.7 (8.0)	30.8^{a} (20.0)	22.0^{b} (18.9)
7	My skin looks unnatural in this under this lighting condition' (yes=0; no=100)	62.7 (28.2)	76.0 (32.2)	75.4 (33.5)	54.2 (34.5)	72.6 (33 .4)	72.4 (34.2)
8	'The light is too 'warm' for a working space' (yes=0; no=100)	70.8 (25.8)	85.3^{a} (18.3)	91.0^{b} (13.8)	76.5 (28.6)	83.8 (21.6)	87.4 (20.9)
9	'The light is too 'cold' for a working space' (yes=0; no=100)	70.9 (24.3)	84.5 (19.4)	87.3^{b} (18.5)	53.5 (36.6)	78.9 (26.0)	84.1^{b} (22.7)
10	'Compared to my usual working conditions, this lighting is…' (better=0 worse=4)	4.0^{d} (0.8)	2.8^{a} (0.8)	2.3^{b} (0.9)	4.6 (0.5)	2.4^{a} (0.9)	2.4^{b} (0.8)
11	'In this lighting environment I could imagine to work for …hours'	1.8 (0.8)	3.2^{a} (2.1)	3.4^{b} (0.8)	1.4 (0.7)	3.5^{a} (2.1)	3.7^{b} (1.8)

Supplemental Table S1: Mean results (± SD) from the visual comfort ratings (11 ratings). The columns show mean values for each lighting condition (DIM=dim light; BL= constant bright light; SSL= self-selected light; per chronotypes (MT=morning type; ET=evening type). ^{a} = differences between dim and bright light; ^{b} = differences between dim and self-selected light; ^{c} = differences between bright and self-selected light; ^{d}= differences between dim light conditions between MT and ET; ($p<0.05$).

Supplemental Figure S1

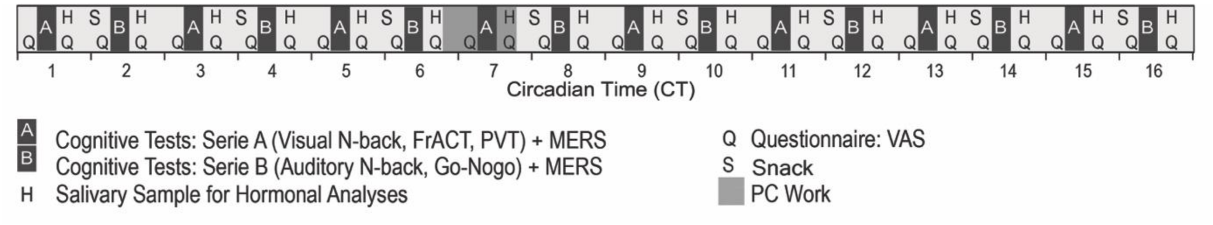

Supplemental Figure S1: Overview of the 16-h study protocol; PVT = Psychomotor Vigilance Test, FrACT = Freiburg Visual Acuity Test, VAS = Visual Analogue Scale, MERS = Mental Effort Rating Scale, PC work = time when subjects were allowed to watch movies on the computer screen or to perform some work.

Supplemental Figure S2

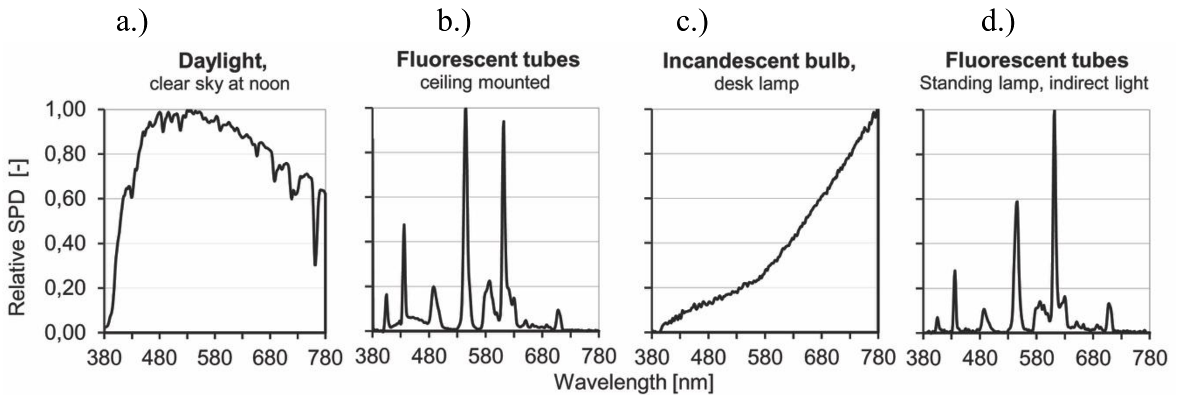

Supplemental Figure S2: Relative spectral power distribution (SPD; relative to the maximum power of each light source) of all light sources in the study room; a): SPD for daylight (measured at a vertical plane at the eye level); b.) ceiling lighting; c.) desk lamp; d.) standing lamp. All recordings were made on a vertical plane, at the eye level of a sitting participant, and in the main direction of view.

Supplemental Figure S3

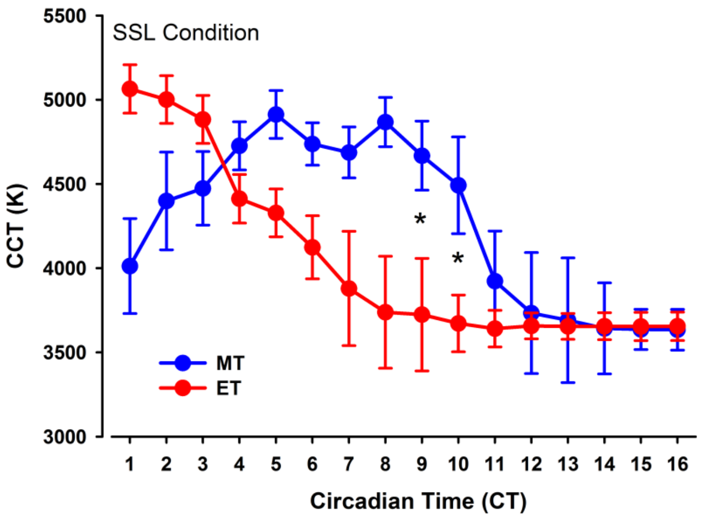

Supplemental Figure S3: The Correlated Color Temperature (CCT; in Kelvin= K) for both chronotypes in the self-selected lighting condition (SSL; means ±SEM per hour; * significant differences between MT and ET at CT 9 and CT 10 (Mann-Whitney U Test and *p*-values adjusted for multiple comparisons). The dynamics are very similar to the curve of the illuminance (see Figure 1 in the main manuscript).

Supplemental Figure S4

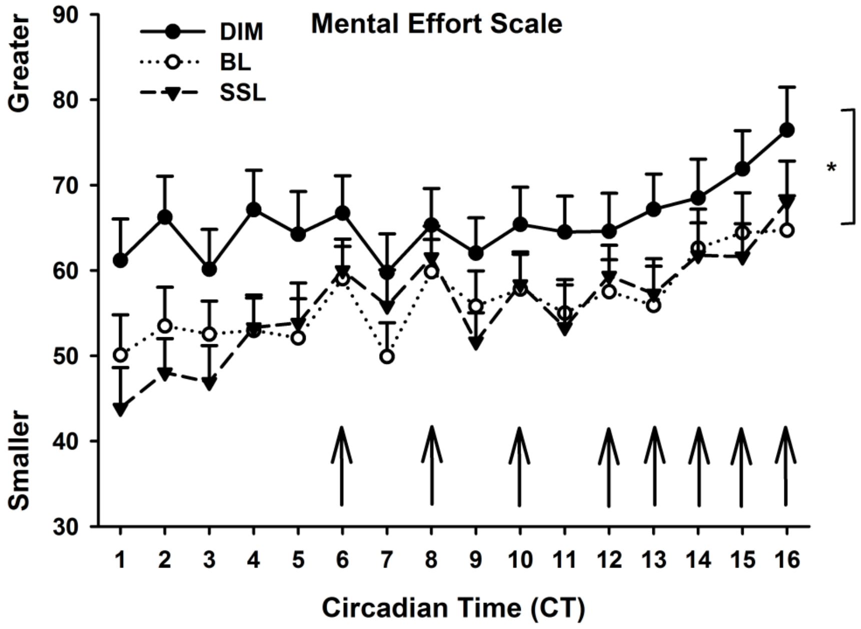

Supplemental Figure S4: The Mental Effort Scale (MERS) for all three lighting conditions (DIM=dim light; BL=constant bright light; SSL=self-selected lighting) across the study duration (CT 1 - CT 16). The arrows indicate where mental load was significantly greater than at the beginning of the study, i.e. at CT 6, 8, 10 and from CT 12-16 (main effect of 'time'; p<0.0001). The mental effort was significantly greater in the DIM condition when compared to both, BL and SSL (main effect of 'condition'; p<0.0001; for more statistics see result section; n=32; mean, SEM).

Supplemental References

1. Horne, J. A. & Östberg, O. A self-assessment questionnaire to determine morningness-eveningness in human circadian rhythms. *International Journal of Chronobiology* **4**, 97-110 (1976).
2. Roenneberg, T., Wirz-Justice, A. & Merrow, M. Life between clocks: daily temporal patterns of human chronotypes. *Journal of Biological Rhythms* **18**, 80-90 (2003).
3. Buysse, D. J., Reynolds, C. F., Monk, T. H., Berman, S. R. & Kupfer, D. J. The Pittsburgh sleep quality index: a new instrument for psychiatric practice and research. *Psychiatry Research* **28**, 193-213 (1989).
4. Taillard, J. *et al.* Time course of neurobehavioral alertness during extended wakefulness in morning- and evening-type healthy sleepers. *Chronobiology International* **28**, 520-527, doi:10.3109/07420528.2011.590623 (2011).
5. Johnson, L. C. Daytime sleepiness in good sleepers: measurement and correlates (eds Broughton JR & Ogilvie RD) 221-229 (Birkhäuser, 1992).
6. Scartezzini, J. L. & Courret, G. Anidolic daylighting systems. *Solar Energy* **73**, 123-135 (2002).
7. Bach, M. The Freiburg visual acuity test-automatic measurement of visual acuity *Optometry & Vision Science, 1996.* **73**, 49 (1996).
8. Eklund, N. & Boyce, P. The development of a reliable, valid and simple office lighting survey. *Journal of Illuminating Engineering Society* **25**, 25-40 (1996).
9. Zijlstra, F. *Efficiency in Work Behavior: A Design Approach for Modern Tools*. (Delft University Press, 1993).
10. Schmidt, C. *et al.* Homeostatic sleep pressure and responses to sustained attention in the suprachiasmatic area. *Science* **324**, 516-519 (2009).
11. Schmidt, C. *et al.* Pushing the limits. Chronotype- and time-of-day modulate working memory-dependent cerebral activity. *Frontiers in Neurology* **6** (2015).
12. Jaeggi, S. M. *et al.* Does excessive memory load attenuate activation in the prefrontal cortex? load-dependent processing in single and dual tasks: functional magnetic resonance imaging study. *NeuroImaging* **19**, 210-225 (2003).
13. Dinges, D. F. & Powell, J. W. Microcomputer analyses of performance on a portable, simple visual RT task during sustained operations. *Behav Res Methods Instrum Comput* **17**, 652-655 (1985).

1
